# Patients visiting the complementary medicine clinic for pain: a cross sectional study

**DOI:** 10.1186/1472-6882-11-36

**Published:** 2011-05-05

**Authors:** Roni Peleg, Orly Liberman, Yan Press, Pesach Shvartzman

**Affiliations:** 1 Department of Family Medicine, Department of Pain and Palliative Care Unit, Siaal Family Medicine and Primary Care Research Center, Ben-Gurion University of the Negev, Beer-Sheva, Israel; 2 Recanati School for Community Health Professions, Ben-Gurion University of the Negev, Beer-Sheva, Israel; 3 Complementary Medicine Clinic, Clalit Health Services - Southern District, Beer-Sheva, Israel

## Abstract

**Background:**

Pain is one of the most common reasons for seeking medical care. The purpose of this study was to characterize patients visiting the complementary medicine clinic for a pain complaint.

**Methods:**

This is a cross-sectional study. The study took place at Clalit Health Services (CHS) complementary clinic in Beer-Sheva, Israel. Patients visiting the complementary clinic, aged 18 years old and older, Hebrew speakers, with a main complaint of pain were included. Patients were recruited consecutively on random days of the month during a period of six months. Main outcome measures were: pain levels, location of pain, and interference with daily activities. Once informed consent was signed patients were interviewed using a structured questionnaire by a qualified nurse. The questionnaire included socio-demographic data, and the Brief Pain Inventory (BPI).

**Results:**

Three-hundred and ninety-five patients were seen at the complementary medicine clinic during the study period, 201 (50.8%) of them met the inclusion criteria. Of them, 163 (81.1%) agreed to participate in the study and were interviewed. Pain complaints included: 69 patients (46.6%) with back pain, 65 (43.9%) knee pain, and 28 (32.4%) other limbs pain. Eighty-two patients (50.3%) treated their pain with complementary medicine as a supplement for their conventional treatment, and 55 (33.7%) felt disappointed from the conventional medicine experience. Eighty-three patients (50.9%) claimed that complementary medicine can result in better physical strength, or better mental state 51 (31.3%). Thirty-seven patients (22.7%) were hoping that complementary medicine will prevent invasive procedures.

**Conclusion:**

Given the high proportion of patients with unsatisfactory pain relief using complementary and alternative medicine (CAM), general practitioners should gain knowledge about CAM and CAM providers should gain training in pain topics to improve communication and counsel patients. More clinical research to evaluate safety and efficiency of CAM for pain is needed to provide evidence based counseling.

## Background

Pain is one of the most common reasons for seeking medical care. It is known to affect general health [1], psychological health [2-4], and economic well-being [5,6].

The prevalence of chronic pain in the general population ranges from 10% to over 40%, depending on the study and definition [7]. Patients with chronic pain use health services up to five times more than patients without pain [8,9]. Although in the last years we have witnessed an impressive improvement in acute and chronic pain treatment still many patients do not get appropriate pain relief. Thus, people use complementary medicine in aim of finding an approach that combines their philosophy of mind-body-spirit and in interest of participating actively in their medical process [10]. One cross-sectional survey of patients with chronic pain disorders conducted in 12 primary care clinics in USA found that 52% reported using complementary and alternative therapies to assist with pain relief [11]. In the subgroup of Canadian adults reporting chronic back pain CAM use was by about 39% [12].

In a study from United Kingdom among patients who had reported having musculoskeletal pain and who had consulted about their pain in primary care in the previous 12 months, 84% had used at least one CAM treatment for pain in the previous year and 65% were current users of CAM. Most CAM treatments were scored by these patients as being helpful, and users indicated that they intended to use again 87% of the CAM treatments they had already used [13]. In another large study [14] the most popular alternative therapies for low back pain noted were spinal manipulation, acupuncture and massage. The American College of Physicians and the American Pain Society have issued joint clinical practice guidelines recommending that clinicians consider acupuncture as one possible treatment option for patients with chronic low back pain who do not have a response to self-care [15,16]. Acupuncture was also found to be effective in improving symptoms of osteoarthritis of the knees, and for chronic headache mainly migraine [17].

In Israel, 6 to 10 percent of Israelies reported using complementary and alternative medicine [18,19] and the rate of use increases [20] . The popularity of complementary medicine in Israel is reflected in the opening of complementary medicine clinics by the different Health Maintenance Organizations (HMOs) and hospitals. Clalit Health Services (CHS), the largest HMO in Israel holds about 40 clinics for complementary medicine throughout the country.

In a study conducted among patients of CHS's complementary medicine clinic in Beer-Sheva, Israel, we found that the most common reason for visiting the complementary clinic were musculoskeletal conditions (47%). Acupuncture and shiatsu massage therapy were the preferred treatments (61% of all treatment provided in the clinic) [21].

As conventional medicine often fails at addressing many patients with a pain experience, and many patients seek other treatment methods. It is important to understand the characteristics of patients attending complementary medicine clinics for a pain complaint.

## Methods

### Design

This is a cross-sectional study.

### Setting

The national health insurance system implemented in Israel in 1995, provides health care to the entire population through non-profit health maintenance organizations (sick funds). The present study was conducted within the framework of the complementary medicine clinic of CHS in Southern Israel, treats annually about 3,000 patients who receive about 15,000 treatments. The treatments offered include acupuncture, reflexology, shiatsu, massage therapy, homeopathy, chiropractic, feldenkreiss, bio feedback, naturopathy, herbal medicine, medical hypnosis and Bach flowers. Patients have a preliminary screening meeting with a physician who recommends the appropriate therapy. The treatments are not included in the national basket of health services, thus patients are required to pay out-of-pocket for these treatments.

### Study population

Patients visiting the complementary medicine clinic, aged 18 year old and older, Hebrew speakers, with a main complaint of pain. Patients were recruited consecutively on random days of the month during a period of six months.

### Data collection

The screening physician recruited patients for the study. Once informed consent was signed patients were interviewed using a structured questionnaire by a qualified nurse. The personal interview with the patient included socio-demographic data, and the Brief Pain Inventory (BPI) questionnaire that had been translated and validated in our previous work [22]. The BPI questionnaire evaluates pain levels on a 0-10 Visual Analogue Scale (VAS), (0-indication no pain; 10-worst possible pain), as well as interference with different aspects of everyday life.

### Statistical analysis

Data was coded and entered into Epidata 2.1 and was analyzed using SPSS 14.0 software (statistical Package for the Social Sciences, SPSS Inc, Chicago).

The study was approved by the institutional review board of Soroka University Medical Center (Approval num 3992).

## Results

Out of 395 patients seen at the complementary medicine clinic during the study period, 201 (50.8%) met the inclusion criteria. Of them, 163 (81.1%) agreed to participate in the study and were interviewed.

The study population characteristics are described in Table 1. The majority were women (66.9%), born in Israel (46.6%), married (73.6%), and the average age was 51.9 ± 15.6. Sixty-four patients (39.3%) were self referred. Fifty one patients (31.3%) were referred by the medical staff and 38 (23.3%) were referred by a friend or family member. Of the patients reported to have chronic diseases (51%) the most common was hypertension with 51.3%, followed by diabetes 19.2%, and heart disease 15.4%.

**Table 1:** Socio-demographic Data of the study population

	N = 163	
	**N**	**%**

**Gender**		

Female	109	66.9

**Age**		

Average ± Std	51.89 ± 15.66	

**Family status**		

Married	120	73.6

**Country of birth**		

Israel	76	46.6

Asia, North Africa	51	31.3

East Europe, former USSR	31	19.0

West Europe, North America, South Africa	5	3.1

**Years in the country (for immigrants)**		

Average ± Std	42.25 ± 14.95	

Range	7-60	

**Years of education**		

1-12	59	44.7%

13-15	32	24.2%

16+	41	31.1%

Average ± Std	13.68 ± 3.21	

Range	2-22	

	**132**	(mis = 31)

**Source of referral**		

Self- initiative	64	39.3

Medical staff	51	31.3

Friend or family member	38	23.3

Other	10	6.2

Sixty-nine patients (46.6%) had a back pain, 65 (43.9%) had a knee pain and 28 (32.4%) other limbs pain. One-hundred-and six patients (68.4%) reported a moderate pain level on average (VAS 4-7), and 28% reported a severe pain level (VAS 8-10). The average level of pain during the last pain attack was 5.6 ± 1.9 points of the VAS and the "conventional" treatment helped relief less than half of pain severity (Table 2).

**Table 2:** Medical Characteristics for Patients with Pain Complaint

	N = 163	
	**N**	**%**

**Pain site**		
Back + waist + hip	71	46.4
Knee	65	42.5
Limbs	50	32.7
Shoulder	38	24.8
Throat and neck	26	17.0
Joints	9	5.9
Head	7	4.6
Buttocks	5	3.3
Unknown	4	2.6
Stomach	3	2.0
	**153**	(mis = 10)
**The average level of pain suffered at the last pain attack (0-no pain, 10- severe pain)**		
0-3	21	13.5
4-7	106	68.4
8-10	28	18.1
Average ± Std	5.57 ± 1.91	
Median	5.00	
	**155**	(mis = 8)
**Present pain level**		
0-3	66	42.6
4-7	72	46.5
8-10	25	16.1
Average ± Std	4.35 ± 2.95	
Median	4.00	
	**163**	
**At the last pain event, did the treatment or medicine helped? (100%=full relief)**		
10-30%	55	35.5
40-70%	52	33.5
80-100%	34	21.9
Average ± Std	48.08 ± 29.49	
Median	50.00	
	**141**	(mis = 22)

Pain impact on daily activities is presented in Table 3. Fifty-eight patients (35.6%) reported that their pain severely influenced their daily routine. Pain caused for 54 (33.1%) patients inability to work or severely interrupted their ability to work. The life impact index was calculated for this group. Pain had high influence on the life impact index for 29 patients (17.8%), (Table 3). The impact of pain on daily activities was evaluated against the socio-demographic characteristics of the study population. In general, older age was found significantly associated with the impact of pain on mood, ability to walk, and relationships with other people (p = 0.05, p = 0.05, p = 0.001 respectively). All other variables, country of birth, gender, religiousness, and education were not found associated with the impact of pain on daily living. The life impact index was also evaluated against all socio-demographic characteristics and was not found significant.

**Table 3:** Pain influence on daily routine (scale of 0-10: 0-no disturbance; 10-highest disturbance)

	N = 163	
	**N**	**%**

**Daily activity**		
0-3	40	24.5
4-7	65	39.9
8-10	58	35.6
Average ± Std	5.83 ± 3.13	
Median	6.00	
	**163**	
**Mood**		
0-3	46	28.2
4-7	59	36.2
8-10	57	35.0
Average ± Std	5.63 ± 3.19	
Median	6.00	
	**162**	(mis = 1)
**Ability to walk**		
0-3	55	33.7
4-7	57	35.0
8-10	51	31.3
Average ± Std	5.03 ± 3.44	
Median	6.00	
	**163**	
**Routine work**		
0-3	41	25.2
4-7	67	41.1
8-10	54	33.1
Average ± Std	5.53 ± 3.15	
Median	6.00	
	**162**	(mis = 1)
**Relationships with other people**		
0-3	107	65.6
4-7	35	21.5
8-10	20	12.3
Average ± Std	2.56 ± 3.19	
Median	1.00	
	**162**	(mis = 1)
**Sleep**		
0-3	56	34.4
4-7	53	32.5
8-10	52	31.9
Average ± Std	5.61 ± 3.41	
Median	6.00	
	**161**	(mis = 2)
**Enjoyment from life**		
0-3	54	33.1
4-7	60	36.8
8-10	47	28.8
Average ± Std	5.17 ± 3.13	
Median	5.00	
	**161**	(mis = 2)
**Life impact (0-low influence, 10-high influence)**		
0-3	35	21.5
3.1-7	99	60.7
7.1-10	29	17.8
Average ± Std	5.00 ± 2.25	
Median	5.14	
	**163**	

The most common symptoms reported in addition to pain (more than one could be marked) were: sleep disorders (55.2%), tiredness (54.6%) and dizziness (21.5%). Eighty-two patients (50.3%) treated their pain with complementary medicine as a supplement for their conventional treatment, and 55 (33.7%) felt disappointed from the conventional medicine experience. Eighty-three patients (50.9%) claimed that complementary medicine can result in better physical strength, or better mental state 51 (31.3%). Thirty-seven patients (22.7%) were hoping that complementary medicine will prevent invasive procedures. Thirty-eight patients (23.3%) experienced, in the past, acupuncture treatment, 27 (16.6%) had reflexology treatment and 18 (11%) shiatsu treatment (Table 4).

**Table 4:** Characteristics of Complementary Medicine Treatments

	N = 163	
	**N**	**%**

**Reason for visiting the complementary medicine clinic (Total may exceed 100% as more than one category could be marked):**		
To complement conventional treatments	82	50.3
Disappointment from conventional medicine	55	33.7
Success of complementary medicine for family member/friend	45	27.6
Complementary medicine is known to be successful for the problem I am suffering from	43	26.4
Complementary medicine does not have side affects	38	23.3
Other	19	11.7
Complementary medicine relate to my problem more seriously	16	9.9
Complementary medicine gives explanations for the problems that I am suffering from	15	9.2
Disappointment from the conventional clinic's staff members	5	3
**How can the conventional treatment be useful (Total may exceed 100% as more than one category could be marked):**		
Physical strength	83	50.9
Mental strength	51	31.3
To avoid invasive treatment	37	22.7
Don't know	32	19.6
Raising awareness for self-treatment	27	16.6
Ease the pain	11	6.7
Annulment of pain	9	5.5
Other	6	3.7
**Have you experienced complementary medicine treatment in the past?**		
Yes	65	43.0
No	86	57.0
**Type of treatment experienced in the past (Total may exceed 100% as more than one category could be marked):**	**151**	(mis = 12)
Acupuncture	38	23.3
Reflexology	27	16.6
Shiatsu	18	11.0
Chiropractic	11	6.7
Other	10	6.1
Herbal medicine	5	3.1
Biofeedback	4	2.5
Homeopathy	3	1.8
Naturopathy	3	1.8

## Discussion

Our findings indicate that approximately half the patients attending the complementary clinic for treatment visited due to a pain complaint, where back pain was the leading cause. The majority were women (66.9%), and the average age was 51.9 ± 15.6. Sixty-four patients (39.3%) were self referred. About two thirds reported a moderate pain level on average (VAS 4-7), and a quarter reported on severe pain level (VAS 8-10). The "conventional" treatments helped relief less than half of pain severity.

Our patients visiting the complementary medicine clinic indicated that the pain impacted their daily routine, caused work disturbance and influenced considerably their daily life. In many cases, people concurrently used complementary medicine with conventional medicine in hope of finding the "magic bullet" cure, though also realizing they need to find other ways to cope and to improve their quality of life [23].

An earlier study (from different countries, including Israel) showed that more women, more educated people and more married people [12,20] are referred to complementary medicine [20,24,25]. Middle age (between 40 to 60-65) was considered a predictor to attending complementary medicine [12,25,26]. In our sample, the average age was 51.9. In a survey by Shmueli and Shoval [18] the typical patient in complementary medicine is older (average age was 57.7). However, this is still considered "middle age", and the difference is probably due to the fact that the population in Beer-Sheva is younger than in the rest of the country.

One of the predictors to attendance to complementary medicine according to the literature is the existence of chronic diseases [24,26,27]. Similar findings were shown in our study, with 51% of patients recruited suffering from a chronic illness.

The main reason for attending complementary medicine clinic among patients in our survey was to supplement conventional medicine treatments (50.3% participants). Moreover, in our study, a third of our study population was referred to the complementary medicine clinic by medical teams. It is known, that only a minority of patients report CAM use to their physicians [27,28]. Thus, there is an increasing need to improve efforts and communication between conventional physicians and complementary therapists [19].

The complementary medicine clinic in Beer Sheva offers a large spectrum of treatment modalities. Complementary medicine includes several methods of treatment, all of which aim to promote health and quality of life [29]. The main reason for nearly half of those attended to the CAM clinic was in aim of seeking treatment for their pain. The most frequent treatment for this complaint was acupuncture [21].

In designing this study, we adopted recommendations to increase the compliance rate for completion of questionnaires [30]. The response rate of 81.1% was achieved by virtue of the data collection method that included personal interview with each patient, using a short questionnaire that carried the logo of the University Research Center, and use of relatively high-motivated study population.

Our study's results in the complementary medicine clinic in Beer-Sheva reflect the characteristics of the patients that used the complementary clinic for their pain. Since the population in Beer-Sheva is younger than in the rest of the country we cannot be certain of their generalization to other countries and health care systems. However, the age difference is not clinical significant and the results were similar to previous studies.

## Conclusions

Patients visiting the complementary medicine clinic indicated that the pain impacted their daily routine, caused work disturbance and influenced considerably their daily life. The most common reasons for patients with a pain complaint visiting the complementary medicine clinic were: as a supplement for conventional treatment, disappointment from conventional medicine and successful previous experience with complementary medicine by a family member or a friend. Given the high proportion of patients with unsatisfactory pain relief using CAM, general practitioners should gain knowledge about CAM and CAM providers should gain training in pain topics to improve communication and counsel patients. More clinical research to evaluate safety and efficiency of CAM for pain is needed to provide evidence based counseling.

We hope that our data can promote understanding the meaning of complementary medicine for painful patients.

## Competing interests

The authors declare that they have no competing interests.

## Authors' contributions

RP conceived of the study, and participated in its design and coordination and helped to draft the manuscript. OL conceived of the study, and participated in its design and coordination and helped to draft the manuscript. YP consultation and study design, helped to draft the manuscript. PS coordinating the study, and participated in its design and helped to draft the manuscript. All authors read and approved the final manuscript.

## Pre-publication history

The pre-publication history for this paper can be accessed here:

http://www.biomedcentral.com/1472-6882/11/36/prepub
